# Up-regulation of Osh6 boosts an anti-aging membrane trafficking pathway toward vacuoles

**DOI:** 10.15698/mic2022.08.783

**Published:** 2022-07-15

**Authors:** Ilham Kadhim, Nazneen Begum, William King, Licheng Xu, Fusheng Tang

**Affiliations:** 1 Department of Biology, University of Arkansas, Little Rock, AR 72204, USA.

**Keywords:** Osh6, PI4P, Golgi, vacuole, longevity

## Abstract

Members of the family of oxysterol-binding proteins mediate non-vesicular lipid transport between membranes and contribute to longevity in different manners. We previously found that a 2-fold up-regulation of Osh6, one of seven yeast oxysterol-binding proteins, remedies vacuolar morphology defects in mid-aged cells, partly down-regulates the target of rapamycin complex 1 (TORC1), and increases the replicative lifespan. At the molecular level, Osh6 transports phosphatidylserine (PS) and phosphatidylinositol-4-phosphate (PI4P) between the endoplasmic reticulum (ER) and the plasma membrane (PM). To decipher how an ER-PM working protein controls vacuolar morphology, we tested genetic interactions between *OSH6* and *DRS2*, whose protein flips PS from the lumen to the cytosolic side of the Golgi, the organelle between ER and vacuoles in many pathways. Up-regulated *OSH6* complemented vacuolar morphology of *drs2*Δ and enriched PI4P on the Golgi, indicating that Osh6 also works on the Golgi. This altered PI4P-enrichment led to a delay in the secretion of the proton ATPase Pma1 to the PM and a rerouting of Pma1 to vacuoles in a manner dependent on the trans-Golgi network (TGN) to late endosome (LE) trafficking pathway. Since the TGN-LE pathway controls endosomal and vacuolar TORC1, it may be the anti-aging pathway boosted by up-regulated Osh6.

## INTRODUCTION

The family of oxysterol-binding proteins is conserved in all eukaryotes. They mediate non-vesicular lipid transport between membranes and thus play critical roles in cell growth and development [1]. Knocking out all seven oxysterol-binding proteins encoded by *OSH* genes in yeast leads to highly fragmented vacuoles and cell death [2]. Late endosomes, the organelle immediately upstream of vacuoles (mammalian lysosomes) in multiple membrane trafficking pathways, are also targets of oxysterol-binding proteins. In *Caenorhabditis elegans*, knocking down the expression of all four oxysterol-binding proteins leads to enlarged late endosome (LE) [3]. In HeLa cells, knocking down the expression of oxysterol-binding protein ORP1L leads to enlarged LE [3]. Since enlarged LE is usually a sign of senescent cells [4], normal functions of oxysterol-binding proteins are thus critical for longevity.

We previously found that a 2-fold up-regulation of Osh6 (P_*ERG6*_-*OSH6*) remedies vacuolar morphology defects in mid-aged cells and extends the replicative lifespan. Interestingly, the level of Osh6 protein in wild type cells declines with age. Moreover, up-regulation of Osh6 partly represses the target of rapamycin complex 1 (TORC1) but requires TORC1 for longevity [5]. This two-way relationship with TORC1 is supported by a recent study on the spatial dissection of TORC1. In yeast cells, the vacuolar TORC1 stimulates protein synthesis and cell growth while the late endosomal TORC1 inhibits autophagy, an anti-aging process through which vacuoles (mammalian lysosomes) degrade damaged and obsolete proteins, lipids, and organelles for longevity [6]. This spatial dissection of TORC1 offers us a new route to explore how Osh6 contributes to longevity.

Osh6 transports phosphatidylinositol-4-phosphate (PI4P) from the plasma membrane (PM) to the endoplasmic reticulum (ER), where Sac1 dephosphorylates PI4P, and in turn transports phosphatidylserine (PS) from the ER to the PM [7]. Other Osh proteins also relay PI4P to Sac1 in the ER but extend the lifespan when depleted from the cell [8]. PS is a unique ligand of Osh6 and its closest paralog Osh7 [7]. Down-regulation of the PS synthesis in ER leads to fragmented vacuoles [9]. PS synthesized in the ER has to be transported to the Golgi and/or other organelles to control vacuolar morphology [10]. Disrupting the flip of PS from the lumen of the Golgi to the cytosolic side also leads to highly fragmented vacuoles [11]. A key enzyme for such flipping is Drs2 [12]. The fact that up-regulated Osh6 promotes vacuole fusion [5] and that Osh6 transports PS and PI4P [7] led us to hypothesize that Osh6 may mediate PS/PI4P transport in intracellular membranes.

In support of the above hypothesis, we found an accumulation of PI4P on the Golgi in cells of P_*ERG6*_-*OSH6*. The accumulation site is likely the trans-Golgi network (TGN) since P_*ERG6*_-*OSH6* mimicked mutants defective in Golgi PS and PI4P trafficking [12] in rerouting a portion of the PM protein Pma1 to vacuoles. Intriguingly, this rerouting of Pma1 was dependent on the TGN-to-LE trafficking pathway. Based on these new findings and other published results, we propose that up-regulation of Osh6 speeds up vesicle trafficking between the TGN and LE by adjusting the local concentration of PI4P and PS on the TGN.

## RESULTS

### *OSH6* genetically interacts with *DRS2*, whose protein works on the Golgi

**Figure 1:**
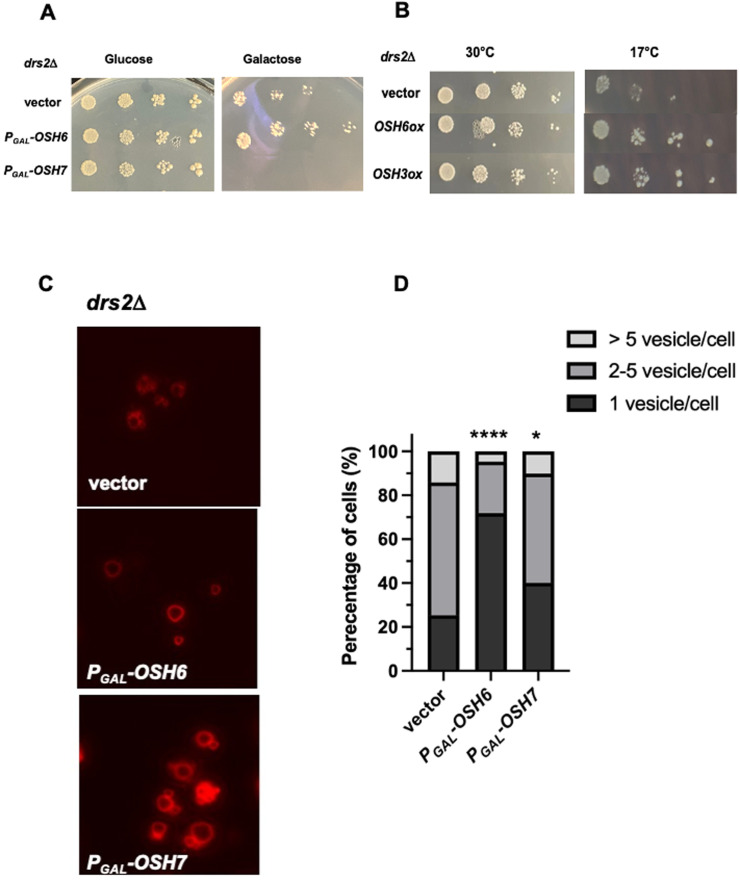
FIGURE 1: *OSH6* genetically interacts with *DRS2*. **(A)** Growth of *drs2*Δ cells with vector, P_*GAL*_-*OSH6* (pCB248), or P_*GAL*_-*OSH7* (pCB247) on SC-URA with glucose or galactose media at 30°C for two days. **(B)** Growth of *drs2*Δ cells with vector, high copy *OSH6* (pCB237), or high copy *OSH3* (pCB238) on SC-URA at 30°C for two days or 17°C for ten days. For A and B, 5 µl of serially diluted cells (0.1 OD/ml for the left) were spotted on the plate and then incubated. **(C)** Vacuolar morphology of *drs2*Δ cells with vector or the indicated plasmid. Overnight cultures were labeled with FM4-64 for one hour and chased at 30°C for three hours and then photographed. **(D)** Quantitative analyses of vacuolar morphology from Fig. 1C. Cells were divided into three categories based on the number of vacuolar vesicles/cell. Sample sizes are 138 for *drs2*Δ (vector), 180 for *drs2*Δ (Pgal-P_*GAL*_-*OSH6*) and 194 for *drs2*Δ (P_*GAL*_-*OSH7*). A one-way ANOVA analysis shows that the fraction of cells with one vesicle/cell of P_*GAL*_-*OSH6* is significantly different from that of vector (p<0.0001). Differences between P_*GAL*_-*OSH7* and wild type is also significant (p=0.012).

To explore how Osh6 affects vacuoles and longevity, we tested potential genetic interactions between *OSH6* and genes involved in the metabolism of PS and PI4P, ligands of Osh6. Drs2 flips PS from the lumen of the Golgi to the cytosolic side and leads to fragmented vacuoles when mutated [11, 12]. We over-expressed *OSH6* and other *OSH* genes in a *drs2*Δ mutant (**Fig. 1A, 1B**). Interestingly, over-expression of *OSH6* by a galactose promoter assisted the growth of *drs2*Δ cells marginally but over-expression of its closest paralog *OSH7* severely delayed the cell growth at 30°C (**Fig. 1A**). At 17°C, over-expression of *OSH6* or *OSH3* by a high copy plasmid complemented the cold sensitivity of *drs2*Δ (**Fig. 1B**). We further checked the vacuolar morphology of the *drs2*Δ mutants with over-expressed *OSH* genes and found that over-expression of *OSH6* significantly promoted vacuole fusion in *drs2*Δ while over-expression of *OSH7* only marginally affected the morphology (**Figs. 1C, 1D**, and Fig. S1A).

Different from their effects on *drs2*Δ, over-expression of *OSH6/7* did not obviously affect the growth of *sac1*Δ (Fig. S1B). For vacuolar morphology, over-expression of *OSH6* did not show an obvious impact on *sac1*Δ (Fig. S1C). The different impact of *OSH6* and *OSH7* genes on *drs2*Δ and *sac1*Δ mutants suggest that Osh6 and Osh7 proteins have their own working locations in addition to their common ER-PM contact site. To search for such unique working places of Osh6, we then monitored the localization of PS and PI4P in cells of different mutants.

### Up-regulation of *OSH6* causes accumulation of PI4P on the Golgi

We first checked the localization of PS by the GFP-Lact-C2 probe, which was already used by many other labs [10, 13]. To use this plasmid, we upregulated the expression of *OSH7* by replacing its endogenous promoter on its chromosome by a short version of the promoter of *ERG6* to obtain P_*ERG6*_-*OSH7* as we did for the up-regulation of *OSH6* [5]. Similar to a previous report [13], up-regulation of Osh6 enriched PS on the PM of small buds (Fig. S2). We did not notice any obvious intracellular accumulations of PS in either P_*ERG6*_-*OSH6* or P_*ERG6*_-*OSH7* cells when compared with wild type cells (Fig. S2). This lack of differences in PS localization seems inconsistent with the genetic interactions between *OSH6* and *DRS2* (**Fig. 1**). This apparent inconsistency is likely due to the limitation of our current assay method for PS localization.

**Figure 2:**
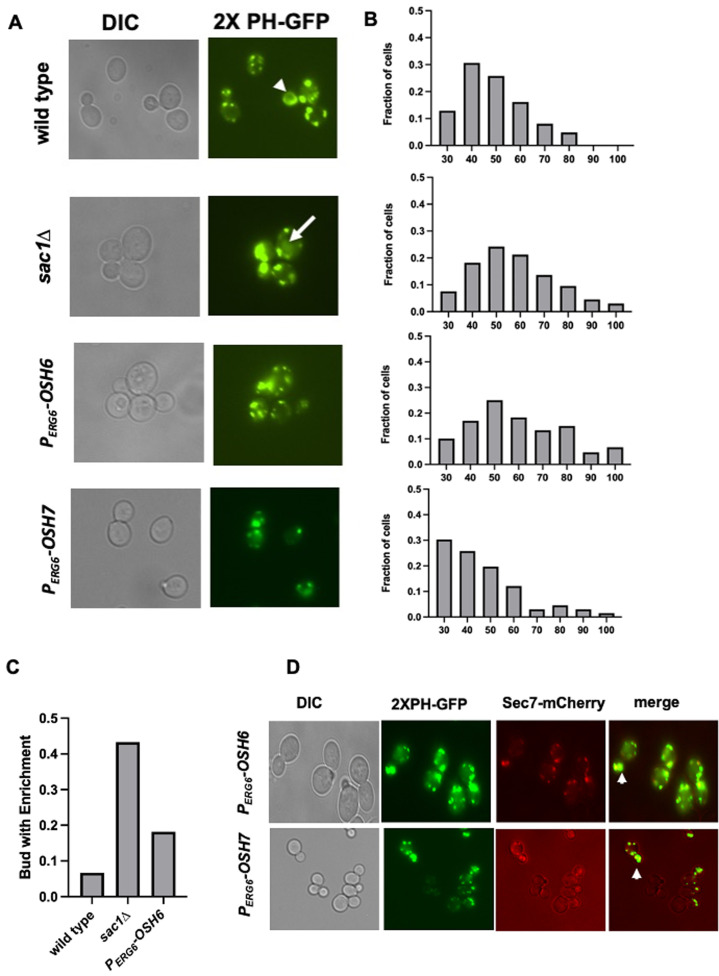
FIGURE 2: Up-regulation of Osh6 traps PI4P on the Golgi. **(A)** Comparison of the localization of PI4P in wild type (BY4742), *sac1*Δ, P_ERG6_-*OSH6* (FTY536), and P_*ERG6*_-*OSH7* (FTY521). The indicated strains were transformed with the 2XPH-OSBP-GFP plasmid, grown to early log phase and photographed. The arrowhead points to a cell with PI4P on the PM in addition to intracellular punctate. The arrow points to a cell with PI4P-decorated circular organelles in *sac1*Δ. **(B)** Distribution of cells with different PI4P intensities. The intensity of PI4P of each cell was measured by the ImageJ software. Then cells were grouped based on the relative PI4P intensity with a ten arbitrary unit (reported by ImageJ) interval (X-axis). The fraction of each group in the whole set is presented on Y-axis. Sample sizes are 61 for wild type, 64 for *sac1*Δ, 83 for P_ERG6_-*OSH6*, and 69 for P_*ERG6*_-*OSH7*. **(C)** Distribution of cells with bud-enriched PI4P in different strains. A cell was counted as ‘bud enriched' if its bud PI4P signal/(bud signal + mother signal) is larger than 0.6. Sample sizes are 31 for wild type, 34 for *sac1*Δ and 35 for P_ERG6_-*OSH6*. Fisher exact test shows that *sac1*Δ is significantly different from wild type (P=0.029) and that *P*_*ERG6*_-*OSH6* is not significantly different from wild type. **(D)** PI4P colocalized with the trans-Golgi network marker Sec7-mCherry. Cells of the Sec7-mCherry integrated version of P_*ERG6*_-*OSH6* (FTY624) and P_*ERG6*_-*OSH7* (FTY625) strains were transformed with the 2XPH-OSBP-GFP plasmid. Early log phase cells of the transformants were photographed under FITC or Texas Red filter. Representative images are shown here. Arrow heads point to areas where PI4P overlapped with Sec7mCherry.

Then we monitored the localization of PI4P. We chose the probe 2XPH-OSBP-GFP because it tends to bind Golgi localized PI4P due to the pH of the cytoplasm [14–16]. Like previously reported, 2XPH-OSBP-GFP visualized PI4P-decorated punctates, typical Golgi structures (**Fig. 2A**). In the wild type, some cells showed plasma membrane PI4P with this probe (see arrowhead-pointed cell in **Fig. 2A**). Expectedly, deletion of Sac1 (*sac1*Δ) accumulated PI4P (**Figs. 2A, 2B**). Interestingly, P_*ERG6*_-*OSH6* accumulated PI4P inside cells (**Figs. 2A, 2B**). P_*ERG6*_-*OSH7* cells also showed punctate PI4P, but with less brightness (**Fig. 2A, 2B**). The fraction of cells carrying high intracellular PI4P levels was much higher in *sac1*Δ and P_*ERG6*_-*OSH6* than that in wild type and P_*ERG6*_-*OSH7* (**Fig. 2B**).

A Fisher Exact test showed that the fraction of cells with high PI4P intensity (>50 AU/cell) in *sac1*Δ (31/64) (p=0.043) and P_*ERG6*_-*OSH6* (42/83) (p=0.016) are significantly higher than that in wild type. Although both P_*ERG6*_-*OSH6* and *sac1*Δ accumulated PI4P, they distribute PI4P differentially. P_*ERG6*_-*OSH6* only showed punctate structures of PI4P, while *sac1*Δ cells also had PI4P on large membrane encircled organelles (arrow-pointed structure in **Fig. 2A**). Moreover, about 1/2 of *sac1*Δ cells showed bud-enriched PI4P, whereas only about 1/5 of P_*ERG6*_-*OSH6* cells showed such an enrichment (**Fig. 2C**). In wild type cells, only about 1/10 of cells had such bud-enriched PI4P. The polarized secretion to the budding tip and small buds depends on dephosphorylation of PI4P by Sac1 [17]. Deletion of Sac1 likely caused an accumulation of PI4P in polarized secretory vesicles. The observation that P_*ERG6*_-*OSH6* had a mild accumulation of PI4P in small buds (**Fig. 2D**) suggests that P_*ERG6*_-*OSH6* has more polarized secretion and/or partial defect in dephosphorylation of PI4P in small budded cells. In support, P_*ERG6*_-*OSH6* cells have more polarized actin cables [5], along which secretory vesicles are transported.

To check whether the punctate PI4P is on the Golgi in P_*ERG6*_-*OSH6*, we integrated the Sec7-mCherry coding DNA into the *URA3* gene [18] in P_*ERG6*_-*OSH6* and P_*ERG6*_-*OSH7* cells and then checked the colocalization of PI4P and Sec7 (a Golgi marker; **Fig. 2D**). In both P_*ERG6*_-*OSH6* and P_*ERG6*_-*OSH7* cells, PI4P did overlap with the Sec7-mCherry (see arrowhead pointed structures in **Fig. 2D**), suggesting that up-regulation of Osh6 enriches PI4P on the Golgi. The higher enrichment of PI4P on the Golgi in P_*ERG6*_-*OSH6* than in P_*ERG6*_-*OSH7* may be a contributor for longevity, since P_*ERG6*_-*OSH7* did not extend the replicative lifespan (data not shown).

### Up-regulation of *OSH6* delays the Pma1 secretion, a function of the Golgi

**Figure 3:**
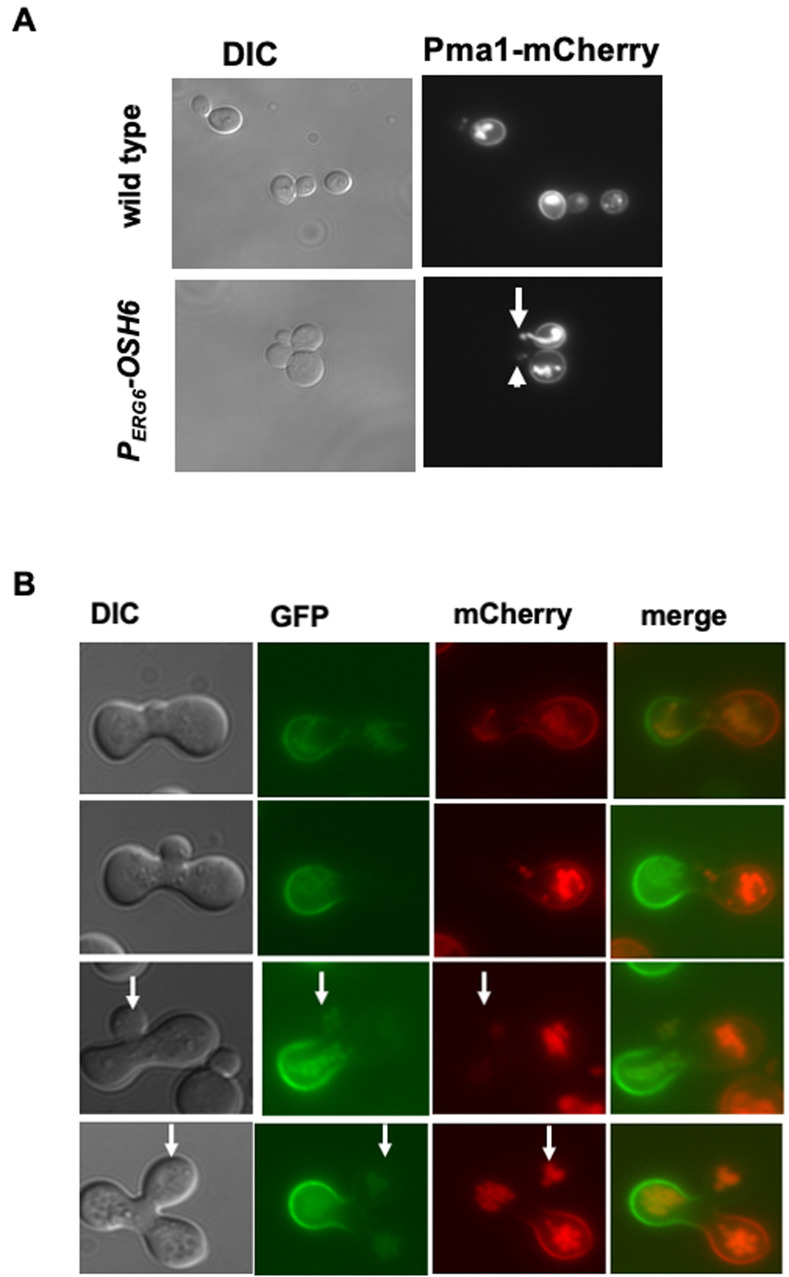
FIGURE 3: Up-regulation of Osh6 delays the secretion of Pma1. **(A)** Comparison of the localization of Pma1-mCherry in wild type (QAY559) and P_*ERG6*_-*OSH6* (FTY518). Cells were grown to early log phase and photographed. The arrow points to vacuole-like structures of intracellular Pma1. The arrowhead points to a bud without normal Pma1 on the PM. **(B)**. Zygote assay. A MATα strain of Pma1-GFP in wild type (HHY103) was mated with a MATa strain of Pma1-mCherry in P_*ERG6*_-*OSH6* (FTY517) and the resulting zygotes were photographed. Zygotes with different sizes of buds were presented from the top row (smallest bud) to the bottom row (largest bud). Arrows point to buds.

Since both PS and PI4P on the Golgi are required for proper secretion of the PM protein Sur7 and Pma1 [12], the accumulation of PI4P on the Golgi in P_*ERG6*_-*OSH6* (**Fig. 2**) may affect the secretion. Thus, we monitored the localization of PM proteins. For this purpose, we constructed P_*ERG6*_-*OSH6* Sur7-GFP and P_*ERG6*_-*OSH6* Pma1-mCherry strains by mating P_*ERG6*_-*OSH6* with Sur7-GFP or Pma1-mCherry strains [19]. Up-regulation of Osh6 (P_*ERG6*_-*OSH6*) did not show obvious effects on the PM Sur7 (Fig. S3), but affected the secretion of Pma1 (**Fig. 3A**). First, P_*ERG6*_-*OSH6* cells accumulated more Pma1 inside cells; almost every cell had intracellular Pma1. The intracellular accumulation sites of Pma1 are likely in vacuoles, since the structure of the Pma1-accumulated organelle in some small-budded cells is very similar to vacuolar segregation structure during budding (see arrow pointed structure in **Fig. 3A**). Second, many small buds of P_*ERG6*_-*OSH6* cells did not show discernible Pma1 (see arrowhead-pointed buds in **Fig. 3A**), suggesting an alteration in secretion and/or endocytosis in the bud.

To double check the effect of up-regulation of Osh6 on Pma1 in small buds, we conducted a zygote assay (**Fig. 3B**). We mated wild type Pma1-GFP with P_*ERG6*_-*OSH6* Pma1-mCherry and then monitored the fluorescence of zygotes. As shown in **Fig. 3B**, the Pma1-GFP signal from the wild type parent arrived at the zygote bud earlier than the Pma1-mCherry signal from the P_*ERG6*_-*OSH6* parent (see arrow-pointed buds in **Fig. 3B**), indicating that up-regulation of Osh6 delays the arrival of Pma1 to the bud PM. Moreover, the intracellular Pma1 in P_*ERG6*_-*OSH6* moved to the other parent and the bud, phenocopying what vacuoles do in such zygote assays [20].

### Decreasing Golgi PI4P abrogates Osh6's effects on Pma1 distribution

**Figure 4:**
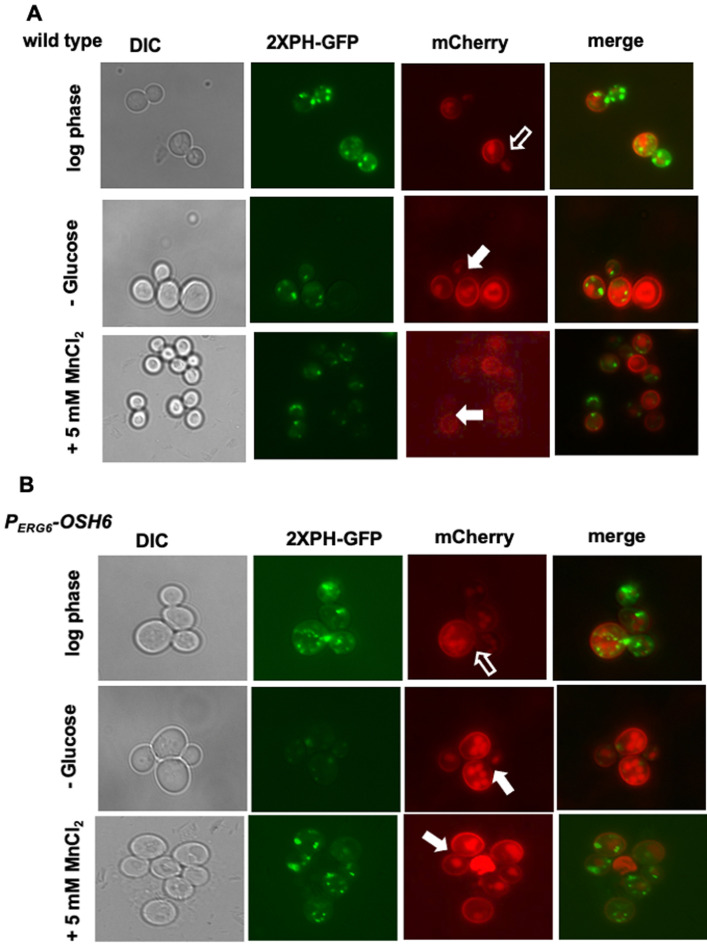
FIGURE 4: Golgi PI4P controls Pma1 secretion. **(A)** The wild type Pma1-mCherry strain (QAY559) was transformed with the PI4P-labeling (2XPH-GFP) plasmid. Transformants were grown in SC-URA with 2% glucose to early log phase (first row), and then starved for glucose for 30 minutes (second row) or treated with 5 mM of MnCl_2_ for 30 min (third row) and photographed. **(B)** The P_*ERG6*_-*OSH6* Pma1mcherry strain (FTY520) was transformed with the PI4P-labeling (2XPH-GFP) plasmid. Transformants were grown in SC-URA to mid-log phase (first row), and then starved for glucose for 30 minutes (second row), or treated with 5 mM of MnCl_2_ (third row). Hollow arrows point to mother-bud neck where the Pma1 signal is not as bright as other PM of its mother cell. Solid arrows point to small or mid-size budded cells with homogenously distributed Pma1 on the PM of the mother cell.

To confirm the Golgi localization of PI4P in P_*ERG6*_-*OSH6*, we monitored the PI4P intensity after glucose starvation (**Fig. 4**). Glucose starvation reallocates the ER-localized Sac1 to the Golgi and prevents the accumulation of PI4P on the Golgi [21]. In line with this previous report, glucose starvation decreased the intensity of PI4P punctate in wild type cells in our assays (compare the GFP pictures of row 1 and row 2 in **Fig. 4A**). Upon glucose starvation, P_*ERG6*_-*OSH6* cells showed a clear decrease in PI4P intensity (compare the GFP pictures of row 1 and row 2 in **Fig. 4B**). In addition to Sac1 action, glucose starvation also changes cytosolic pH and decreases the binding of Golgi PI4P by PH domains [16]. Both possibilities (Sac1, pH) support that PI4P-decorated punctates in P_*ERG6*_-*OSH6* cells are Golgi structures.

Along with the decrease of the Golgi PI4P levels, the secretion pattern of Pma1 was also changed upon glucose starvation. In log phase cells grown in a medium with 2% glucose, the Pma1-mCherry signal on the PM of the mother-bud neck is not as bright as other areas of PM of the mother cell (see hollow arrow pointed areas in wild type (**Fig. 4A**) and P_*ERG6*_-*OSH6* (**Fig. 4B**)). Glucose starvation made Pma1 homogenously distributed on the mother PM (see solid arrow pointed cells in **Fig. 4**). Moreover, we found that treating yeast cells with 5 mM MnCl_2_ also decreased the Golgi PI4P level (**Fig. 4**) and simultaneously altered the secretion of Pma1 similar to what glucose starvation did (**Fig. 4**). These observations further confirm that up-regulation of Osh6 causes accumulation of PI4P on the Golgi.

### The effects of up-regulation of *OSH6* rely on the Golgi-to-LE trafficking

**Figure 5:**
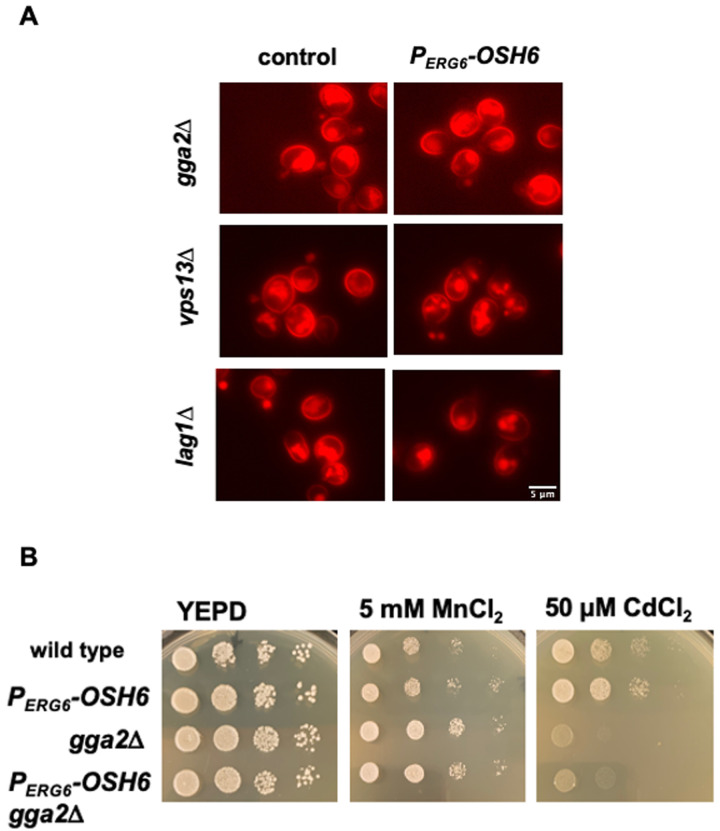
FIGURE 5: Up-regulated Osh6 works through the TGN-LE trafficking pathway. **(A)** The Pma1-mCherry distribution in the indicated strains. The respective control for each indicated mutant is shown on the left with Pma1-mCherry. P_*ERG6*_-*OSH6* side means P_*ERG6*_-*OSH6* Pma1-mCherry carrying the indicated mutations listed on the left. Early log phase cells grown in YEPD were photographed. **(B)** Comparison of growth on metal-containing media. Five μl of 10-fold serially diluted cells (starting from the left 0.1 OD_600_/ml) were spotted on YEPD or YEPD with the indicated salts and grown at 30°C for two days.

While multiple pathways transport post-Golgi vesicles to vacuoles, the pathway that depends on the level of Golgi PI4P is the TGN to LE (TGN-LE) pathway (also termed the CPY pathway) [22]. To test whether the TGN-LE pathway is used by Osh6 in rerouting Pma1 to vacuoles, we checked the impact of Gga2, which binds Golgi PI4P and catalyzes the formation of the CPY vesicles [22], and Vps13, which recycles materials from LE back to the Golgi [23] on the Pma1 localization in P_*ERG6*_-*OSH6* cells (**Fig. 5**). As a control, we also tested the impact of Lag1, a ceramide synthase working in the ER [24]. In the double mutant P_*ERG6*_-*OSH6 gga2*Δ or P_*ERG6*_-*OSH6 vps13*Δ, the Pma1 localization was similar to that in the corresponding *gga2*Δ or *vps13*Δ single mutant, where the intracellular Pma1 is dispersed to almost the whole cytoplasm (**Fig. 5A**). Similar to the trafficking of Pma1, a growth phenotype of P_*ERG6*_-*OSH6 gga2*Δ also followed the pattern of the *gga2*Δ single mutant; P_*ERG6*_-*OSH6 gga2*Δ was as resistant as *gga2*Δ to 5 mM MnCl_2_ and as sensitive as *gga2*Δ to 50 µM CdCl_2_ (**Fig. 5B**). The dependency of Osh6 on Gga2 supports that Osh6 works through the TGN-LE pathway. Different from *gga2*Δ or *vps13*Δ mutants, the P_*ERG6*_-*OSH6 lag1*Δ double mutant behaved similar to P_*ERG6*_-*OSH6* in enriching Pma1 into certain organelles (**Fig. 5A**).

### Up-regulation of *OSH6* has a unique role in restoring cell growth

**Figure 6:**
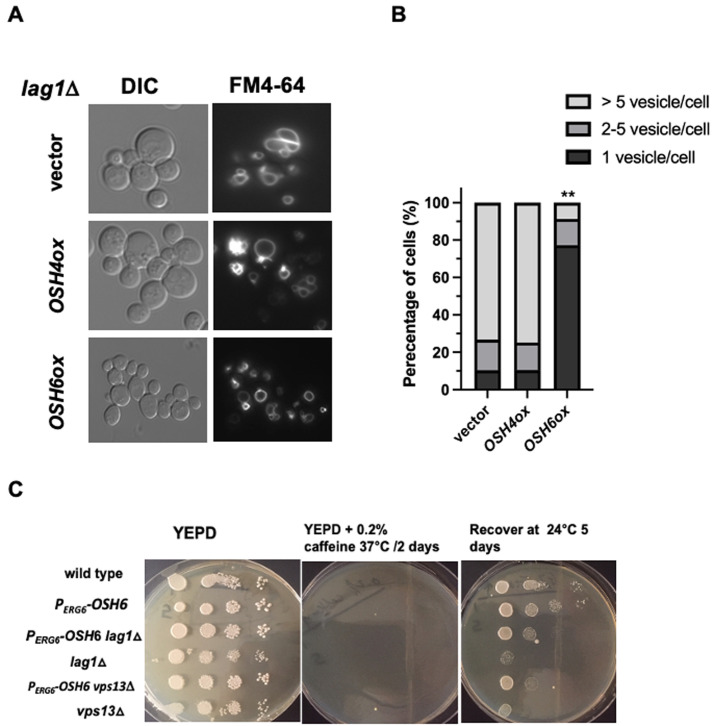
FIGURE 6: Up-regulation of *OSH6* complements defects of *lag1*Δ. **(A)** Comparison of vacuole morphology of *lag1*Δ with the indicated plasmids listed on the left. Vector: YEp24, *OSH4*ox: YEp24-*OSH4*; *OSH6*ox: YEp24-*OSH6*. Log phase cells were labeled with FM4-64 for one hour and chased for three hours at 30°C and then photographed. **(B)** Quantitation of cells with different categories of vacuoles. Sample sizes are 98 for *lag1*Δ(vector), 105 for *lag1*Δ(*OSH4*ox) and 102 for *lag1*Δ (*OSH6*ox). A one-way ANOVA shows that the fraction with one vacuolar vesicle/cell of *lag1*Δ (*OSH6*ox) is significantly higher than that of *lag1*Δ (vector) (p< 0.0001). **(C)** Recovery of cell growth after caffeine and high temperature arrest. Overnight cultures of wild type (BY4742), P_*ERG6*_-*OSH6* (FTY373), *lag1*Δ, P_*ERG6*_-*OSH6 lag1*Δ (FTY527), *vps13*Δ, and P_*ERG6*_-*OSH6 vps13*Δ (FTY534) were serially diluted by 10-fold (starting at 0.1 OD_600_/ml from the left). Five µl of serial diluted cells of the indicated strains were spotted on YEPD (left) and YEPD + 0.2% caffeine plate (middle) and incubated at 37°C for two days. Then the caffeine plate was incubated at room temperature (24°C) for five days.

Pma1 is secreted to the PM by complexing with sphingolipids carrying very long chain fatty acids (VLCFA) in the ER and the Golgi [25]. Since sphingolipids with VLCFA are also required for functions of vacuolar ATPase and vacuolar morphology [26, 27], we studied interactions between Osh6 and Sur4, which synthesizes VLCFA and controls vacuolar morphology via the TGN-LE trafficking step [28], and Lag1, which incorporates VLCFA into ceramide, the precursor for sphingolipids. Over-expressing *OSH6* by a high copy plasmid restored vacuole fusion in *lag1*Δ and *sur4*Δ while over-expression of *OSH4* did not show such restoration (**Fig. 6A** and Fig. S4). The observations that high levels of Osh6 down-regulated the secretion of Pma1 (**Fig. 3**) while promoted vacuole fusion in mutants (*lag1*Δ and *sur4*Δ defective in enzymes in the ER (**Figs. 6A, 6B**, and Fig. S4) further support the idea that Osh6 works on the Golgi.

To further test the genetic interactions between *OSH6* and *LAG1*, we tested growth phenotype of the P_*ERG6*_-*OSH6 lag1*Δ double mutant on caffeine plates (**Fig. 6C**). Caffeine inhibits TORC1 in a manner similar to rapamycin [29]. Shocking cells by 0.2% caffeine at 37°C for two days led to growth arrest (see middle panel of **Fig. 6C**). The growth was recovered after transferring this plate to room temperature (24°C) and incubating for five days (see row 1 and row 2 in **Fig. 6C**). Although *lag1*Δ showed a weak recovery, the P_*ERG6*_-OSH6 *lag1*Δ double mutant recovered 10- to 100-fold better than *lag1*Δ did (rows 3 and 4 in **Fig. 6C**). Different from its effects on *lag1*Δ, P_*ERG6*_-*OSH6* only very mildly recovered the growth of a mutant missing Vps13, which facilitates the TGN-LE trafficking (rows 5 and 6 in **Fig. 6C**). The recovery of growth after TORC1 repression requires TORC1 activation to stimulate protein synthesis as well as TORC1 repression to relieve the inhibition on microautophagy, which affects vacuolar morphology [30]. Since LE TORC1 inhibits autophagy including microautophagy and vacuolar TORC1 stimulates cell growth [6], we interpret the recovery of growth of the P_*ERG6*_-*OSH6 lag1*Δ mutant (**Fig. 6C**) and the promotion of vacuole fusion of Osh6 in *lag1*Δ and *sur4*Δ (**Figs. 6A, 6B**, and Fig. S4) as that up-regulation of Osh6 differentially affects the endosomal TORC1 and vacuolar TORC1 by altering the TGN-LE trafficking step.

## DISCUSSION

While Osh6 and its homologs work on multiple organelles including the ER, the PM, secretory vesicles, and mitochondria [7, 13, 31, 32], it differs from other Osh proteins in its effects on longevity. *OSH6* extends the replicative lifespan when up-regulated, but other *OSH* genes (*OSH1, OSH2, OSH3, OSH4*) extend the lifespan when deleted from the genome [5, 8]. The unique roles of Osh6 and the critical contribution of the TGN- LE trafficking to pro-longevity pathways [6] led us to propose that Osh6 accelerates the TGN-LE vesicle trafficking and differentially affects endosomal TORC1 and vacuolar TORC1 to increase longevity.

### The TGN is a working place of Osh6

Our phenotypic and subcellular localization analyses along with other group's results on Osh6 and PI4P suggest that a subset of Osh6 works on the TGN. Although both P_*ERG6*_-*OSH6* and *sac1*Δ accumulated PI4P inside cells, P_*ERG6*_-*OSH6* caused an enrichment of PI4P on Golgi-like punctate while in *sac1*Δ cells PI4P could also be found on other membranes that gave a faint signal (see arrow-pointed cell in **Fig. 2A**). In log phase cells, intracellular PI4P localizes to the Golgi membrane due to the function of the PI 4 kinase Pik1[33]. In *sac1*Δ cells, PI4P also localizes to other organelles including endosomal/vacuolar membranes and leads to a large vacuole with multiple invaginations [34] (also see Fig. S1C). Different from *sac1*Δ cells, P_*ERG6*_-*OSH6* cells have normal vacuolar morphology, two to five vacuolar vesicles per cell for most cells [5]. Thus, P_*ERG6*_-*OSH6* could not accumulate PI4P on endosomal/vacuolar membranes. Indeed, PI4P in P_*ERG6*_-*OSH6* overlapped with the Golgi marker Sec7 (**Fig. 2D**). The localization data of Osh6 from Orin's lab show that Osh6 can work on TGN. A chromosomal version of GFP-tagged Osh6 with its endogenous promoter localizes to the PM and intracellular organelles in small buds [35]. During yeast cell division, organelles such as ER, late Golgi elements, and vacuoles are transported to buds at an early stage of budding [20, 36, 37]. Since ER hosts Sac1 and degrades PI4P, we conclude that the enriched PI4P in small buds of P_*ERG6*_-*OSH6* (**Fig. 2C**) is a sign of Osh6 working on late Golgi elements. In line with this conclusion, in P_*ERG6*_-*OSH6* cells a portion of Pma1 was rerouted to vacuoles (**Fig. 3**), phenocopying mutants missing Drs2 or Osh4, two proteins working on the TGN [12].

### Osh6 promotes TGN-LE transport to extend the lifespan

**Figure 7:**
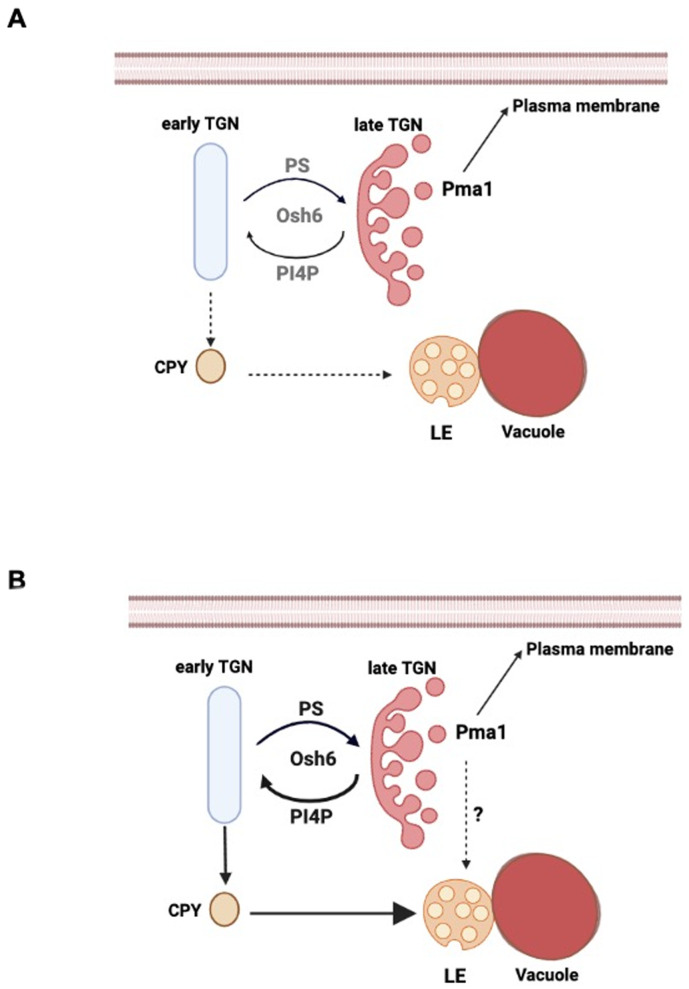
FIGURE 7: A working model for Osh6's role in TGN-LE trafficking. **(A)** A summary of the maturation of the trans-Golgi network (TGN) and post-Golgi trafficking. The formation of Pma1-vesicles for secretion occurs at late cisternae of the TGN while vesicles carrying carboxylpeptidase Y (CPY) or other vacuolar hydrolases to late endosome (LE) occurs in early cisternae of the TGN [41] (see text for detail). **(B)** A hypothetical action of up-regulated Osh6. Up-regulation of Osh6 transports more PI4P to the early cisternae of the TGN, which facilitates the TGN-to-LE trafficking since the formation of the CPY-carrying vesicles depends on Gga2-PI4P binding. The accelerated TGN-LE trafficking refurbishes fresh lipids to LE and vacuoles and thus support vacuolar functions. As a side effect, transporting PI4P out of the late cisternae of TGN delays the secretion of Pma1 and leads to the rerouting of Pma1 to LE and vacuoles.

Three lines of evidence suggest that elevated Osh6 promotes TGN-LE membrane trafficking as depicted in **Fig. 7**. First, P_*ERG6*_-*OSH6* caused an enrichment of PI4P on the Golgi (**Fig. 2**) and this relied on the TGN-LE protein Gga2, which binds Golgi PI4P for its effects (**Fig. 5**).

Second, over-expression of *OSH6* restores vacuole fusion of mutants defective in TGN-LE trafficking. Deletion of Sur4 compromises the TGN-LE trafficking but does not affect the TGN-vacuole pathway [28]. In this work, we observed that over-expression of *OSH6* but not *OSH4* restored vacuole fusion in *sur4*Δ (Fig. S3) and *lag1*Δ (Fig. 4), cells which are defective in incorporating Sur4's product into sphingolipids. The specific growth recovery effect of P_*ERG6*_-*OSH6* on *lag1*Δ (**Fig. 6**) suggests that the TGN-LE trafficking step is the most likely route for Osh6 to control vacuolar morphology and TORC1.

Third, the effect of up-regulation of Osh6 on Pma1 localization is dependent on Gga2 and Vps13, key players in the TGN-LE transport (**Fig. 5**). Moreover, *VPS13* and *OSH6* share two genetic interaction partners, *CSG2* and *VPS35,* and *GGA2* also interacts with *CSG2* [38, 39]. Csg2 is required for the synthesis of mannosyl-inositol-phosphorylceramide, a complex sphingolipid. Vps35 is required for the recycling of vesicles from endosomes back to the TGN and genetically interacts with Tor1 [40]. Thus, a simple model reconciling all phenotypes of P_*ERG6*_-*OSH6* is that elevated Osh6 accelerates the TGN-LE transport and in turn LE-vacuole transport (**Fig. 7B**). Such acceleration limits the membrane size of LE so that LE can only host minimal endosomal TORC1.

### Osh6 may accelerate TGN-LE transport by adjusting the local PS and PI4P concentrations of the early TGN

Osh6's two lipid ligands, PS and PI4P, are both crucial for the formation of post-Golgi vesicles and post-Golgi membrane trafficking. Upon maturation of the trans-Golgi cisternae, vesicles carrying Pma1 and other cargoes are budded from the late TGN cisternae and transported to the PM (summarized in **Fig. 7A**). The Pma1-vesicles require balanced PI4P and PS [12]. During the process of TGN maturation, the early cisternae of the TGN forms vesicles destined to LE [41]. The formation of TGN-LE vesicles depends on Gga2-PI4P interactions [22]. In active growing cells, the TGN-LE transport is very slow [42]. This slow rate is likely caused by lack of PI4P on the early TGN cisternae, since the majority of Golgi PI4P is used for the formation of secretory vesicles and is consumed by Osh4 and other Osh proteins during secretion [12, 17, 31].

Elevated Osh6 could accelerate the TGN-LE transport by its PI4P/PS swapping activity (**Fig. 7B**). The formation of vesicles toward LE requires PI4P [22]. Moreover, PS on LE needs to be transported back to the TGN by the LE-TGN retrograde pathway [43, 44], since elevated PS or PS/phosphatidylethanolamine (PE) ratio on LE and vacuolar membranes leads to fragmented vacuoles [45]. Osh6's working on TGN may transport PI4P to the early TGN cisternae and remove PS from that area (dark arrows **in Fig. 7B**). A support for such removal of PS is the observation that over-expression of *OSH6* complemented defects of *drs2*Δ (**Fig. 1**). This complementation suggests that Osh6 can glean PS for some essential Golgi functions that are normally achieved by Drs2-flipped PS. The potential PI4P/PS swapping on the TGN facilitates both the formation of vesicles toward LE and the LE-to-TGN retrograde trafficking. Such elevated retrograde trafficking also leads to a decrease of LE membrane and thus endosomal TORC1. Testing this model in future studies would reveal detailed mechanistic links between TGN-LE trafficking and endosomal/vacuolar TORC1 activities and hence provide insights on how oxysterol-binding proteins control TORC1 and longevity in other organisms.

## MATERIALS AND METHODS

### Strains, plasmids, yeast manipulations, and media

All yeast strains (listed in Table S1) are derivatives of BY4742. The PS-labeling GFP-Lact-C2 plasmid was kindly provided by Drs. Gregory Fairn and Vanina Zaremberg. The PI4P-labeling 2XPH-OSBP-GFP plasmid pPGK1303 was kindly provided by Dr. Christina Mitchell. OSH plasmids (pCB237 (Yep24-OSH6), pCB238 (Yep24-OSH3), pCB241 (Yep24-OSH4), pCB247 (P_GAL_-OSH7), pCB248 (P_GAL_-OSH6)) were kindly provided by Dr. Christopher Beh. Construction of P_*ERG6*_-*OSH7* is described below. Double and triple mutants of P_*ERG6*_-*OSH6* carrying Pma1-mCherry or Sur7-GFP were constructed by standard yeast mating of P_*ERG6*_-*OSH6* with Pma1-mCherry, Sur7-GFP strains provided by Dr. Mara Duncan [19], induction of meiosis, and tetrad dissection. Standard yeast media YEPD, yeast extract (1%)-peptone (2%)-dextrose (2%), was used unless otherwise stated.

### Construction of P*_ERG6_-OSH7* mutants

The strategies for constructing previously described P_*ERG6*_-*OSH6* mutants [5] were employed to construct the P_*ERG6*_-*OSH7* in this study. The P_*ERG6*_-*OSH7* strain was constructed by switching the endogenous promoter of *OSH7* with the promoter of *ERG6* via *in vivo* recombination. The promoter of *ERG6* on the plasmid pRS316-ERG6 [46] was PCR-amplified by the primer Perg6OSH7up (5′TATCAGTATATTATAGGAATGTTAATTCGCTCGTGCGTACTAATTTTTGATTCGGTAATCTCC) and Perg6OSH7 down (5′ACTGTTTGTTAAAGAAGGTATATTCTTTAGTTTATTGAGAGCCATCATCTTATGCTGCCTACT). This PCR-amplified fragment contained the *URA3* gene followed by the basal *ERG6* promoter flanked by sequences homologous to the *OSH7* promoter. This fragment was transformed into BY4742. Verification of the correct insertion-replacement was confirmed by PCR with primers RCERG6up (5'ATAGTTCGGGTGTTTT) and RCosh7down (5'TGATCTGTTCTTCATG). A 1.5 kb PCR-amplified fragment was used as the diagnostic band for the P_*ERG6*_-*OSH7* replacement.

### PS, PI4P, and vacuole labeling

A plasmid expressing the GFP-Lact-C2 fusion protein, which binds PS [10] or a plasmid expressing PI4P-binding marker proteins (pPGK1303/PH-OSBP-GFP) [15] was transformed into the wild type BY4742 or different mutant strains. Transformation was done with the LiAc protocol following all the steps listed in [47]. If the mutant strain carried the *URA3*-P_*ERG6*_ promoter, the *URA3* marker was replaced by a Kanamycin-resistance marker with *BamHI*-linearized M3927 obtained from Addgene [48] before PS and PI4P assay. Transformants were grown in SC-URA liquid media at 30°C to early log phase (OD_600_ between 0.2 to 0.4). Cells were observed under a 90i eclipse microscope and photographed under the FITC filter for GFP and Texas Red filter for mCherry. The resulting pictures were imported to the ImageJ software to report the fluorescent intensity of the region of interest (bud, mother) that was drawn manually. Intensities obtained for each cell were used for distribution analyses.

Vacuoles were labeled by FM4-64 and chased as described before [49].

### The co-localization of PI4P with the Golgi marker Sec7-mCherry

The *URA3*::*Sec7*-mCherry DNA linearized by *XcmI* and *BstB1* from YIplac211-*SEC7*-mCherry2Bx [18] was transformed into *URA3*-P_*ERG6*_-*OSH6* (FTY373) and *URA3*-P_*ERG6*_-*OSH7* (FTY437) cells. The resulting transformants were selected on SC+URA+5-fluroorotic acid plate. After confirming the lack of growth on SC-URA plates, the Sec7-mCherry version of P_*ERG6*_-*OSH6* (FTY624) and P_*ERG6*_-*OSH7* (FTY625) strains were transformed by the plasmid carrying the PI4P-binding probe (2XPH-OSBP-GFP). The resulting transformants were grown to early log phase in SC-URA and then photographed under the FITC (for PI4P) and Texas Red (for Sec7) filters.

### Image quantitation and statistical analyses

All the pictures were analyzed by the ImageJ software to measure the fluorescent intensity of the region of interest (bud, mother) that was drawn. For quantitative analysis, we used Graph Pad Prism 8 software to generate all the graphs in this study. The significance of the difference in the mean values was determined by 1 WAY ANOVA that was applied on all the data by using Tukey's multiple comparisons test, all those tests indicated P value < 0.0001 was considered as significant. Also, other statistics analysis such as Fisher exact test was conducted to judge whether the fraction of cells with bud-enriched PI4P is significant between two samples.

## SUPPLEMENTAL MATERIAL

Click here for supplemental data file.

All supplemental data for this article are available online at https://www.microbialcell.com/researcharticles/2022a-kadhim-microbial-cell/.

## Abbreviations:

ER: – endoplasmic reticulum,
LE: – late endosome,
PI4P: – phosphatidylinositol-4-phosphate,
PM: – plasma membrane,
PS: – phosphatidylserine,
TGN: – trans-Golgi network,
TORC: – target of rapamycin complex.

## References

[1] Pietrangelo A, Ridgway ND (2018). Bridging the molecular and biological functions of the oxysterol-binding protein family.. Cell Mol Life Sci.

[2] Beh CT, Rine J (2004). A role for yeast oxysterol-binding protein homologs in endocytosis and in the maintenance of intracellular sterol-lipid distribution.. J Cell Sci.

[3] Kobuna H, Inoue T, Shibata M, Gengyo-Ando K, Yamamoto A, Mitani S, Arai H (2010). Multivesicular body formation requires OSBP-related proteins and cholesterol.. PLoS Genet.

[4] Birnbaum A, Sodders M, Bouska M, Chang K, Kang P, McNeill E, Bai H (2021). FOXO Regulates Neuromuscular Junction Homeostasis During Drosophila Aging.. Front Aging Neurosci.

[5] Gebre S, Connor R, Xia Y, Jawed S, Bush JM, Bard M, Elsalloukh H, Tang F (2012). Osh6 overexpression extends the lifespan of yeast by increasing vacuole fusion.. Cell Cycle.

[6] Hatakeyama R, Péli-Gulli MP, Hu Z, Jaquenoud M, Garcia Osuna GM, Sardu A, Dengjel J, De Virgilio C (2019). Spatially Distinct Pools of TORC1 Balance Protein Homeostasis.. Mol Cell.

[7] Maeda K, Anand K, Chiapparino A, Kumar A, Poletto M, Kaksonen M, Gavin AC (2013). Interactome map uncovers phosphatidylserine transport by oxysterol-binding proteins.. Nature.

[8] Huang J, Mousley CJ, Dacquay L, Maitra N, Drin G, He C, Ridgway ND, Tripathi A, Kennedy M, Kennedy BK, Liu W, Baetz K, Polymenis M, Bankaitis VA (2018). A Lipid Transfer Protein Signaling Axis Exerts Dual Control of Cell-Cycle and Membrane Trafficking Systems.. Dev Cell.

[9] Kannan M, Lahiri S, Liu LK, Choudhary V, Prinz WA (2017). Phosphatidylserine synthesis at membrane contact sites promotes its transport out of the ER.. J Lipid Res.

[10] Ganesan S, Sosa Ponce ML, Tavassoli M, Shabits BN, Mahadeo M, Prenner EJ, Terebiznik MR, Zaremberg V (2019). Metabolic control of cytosolic-facing pools of diacylglycerol in budding yeast.. Traffic.

[11] Furuta N, Fujimura-Kamada K, Saito K, Yamamoto T, Tanaka K (2007). Endocytic recycling in yeast is regulated by putative phospholipid translocases and the Ypt31p/32p-Rcy1p pathway.. Mol Biol Cell.

[12] Hankins HM, Sere YY, Diab NS, Menon AK, Graham TR (2015). Phosphatidylserine translocation at the yeast trans-Golgi network regulates protein sorting into exocytic vesicles.. Mol Biol Cell.

[13] D'Ambrosio JM, Albanèse V, Čopič A (2019). Following Anterograde Transport of Phosphatidylserine in Yeast in Real Time.. Methods Mol Biol.

[14] Levine TP, Munro S (2002). Targeting of Golgi-specific pleckstrin homology domains involves both PtdIns 4-kinase-dependent and -independent components.. Curr Biol.

[15] Wiradjaja F, Ooms LM, Tahirovic S, Kuhne E, Devenish RJ, Munn AL, Piper RC, Mayinger P, Mitchell CA (2007). Inactivation of the phosphoinositide phosphatases Sac1p and Inp54p leads to accumulation of phosphatidylinositol 4,5-bisphosphate on vacuole membranes and vacuolar fusion defects.. J Biol Chem.

[16] Shin JJH, Liu P, Chan LJ, Ullah A, Pan J, Borchers CH, Burke JE, Stefan C, Smits GJ, Loewen CJR (2020). pH Biosensing by PI4P Regulates Cargo Sorting at the TGN.. Dev Cell.

[17] Ling Y, Hayano S, Novick P (2014). Osh4p is needed to reduce the level of phosphatidylinositol-4-phosphate on secretory vesicles as they mature.. Mol Biol Cell.

[18] Day KJ, Casler JC, Glick BS (2018). Budding Yeast Has a Minimal Endomembrane System.. Dev Cell.

[19] Lang MJ, Martinez-Marquez JY, Prosser DC, Ganser LR, Buelto D, Wendland B, Duncan MC (2014). Glucose starvation inhibits autophagy via vacuolar hydrolysis and induces plasma membrane internalization by down-regulating recycling.. J Biol Chem.

[20] Weisman LS, Wickner W (1988). Intervacuole exchange in the yeast zygote: a new pathway in organelle communication.. Science.

[21] Piao H, MacLean Freed J, Mayinger P (2012). Metabolic activation of the HOG MAP kinase pathway by Snf1/AMPK regulates lipid signaling at the Golgi.. Traffic.

[22] Demmel L, Gravert M, Ercan E, Habermann B, Müller-Reichert T, Kukhtina V, Haucke V, Baust T, Sohrmann M, Kalaidzidis Y, Klose C, Beck M, Peter M, Walch-Solimena C (2008). The clathrin adaptor Gga2p is a phosphatidylinositol 4-phosphate effector at the Golgi exit.. Mol Biol Cell.

[23] De M, Oleskie AN, Ayyash M, Dutta S, Mancour L, Abazeed ME, Brace EJ, Skiniotis G, Fuller RS (2017). The Vps13p-Cdc31p complex is directly required for TGN late endosome transport and TGN homotypic fusion.. J Cell Biol.

[24] Megyeri M, Prasad R, Volpert G, Sliwa-Gonzalez A, Haribowo AG, Aguilera-Romero A, Riezman H, Barral Y, Futerman AH, Schuldiner M (2019). Yeast ceramide synthases, Lag1 and Lac1, have distinct substrate specificity.. J Cell Sci.

[25] Gaigg B, Timischl B, Corbino L, Schneiter R (2005). Synthesis of sphingolipids with very long chain fatty acids but not ergosterol is required for routing of newly synthesized plasma membrane ATPase to the cell surface of yeast.. J Biol Chem.

[26] Chung JH, Lester RL, Dickson RC (2003). Sphingolipid requirement for generation of a functional v1 component of the vacuolar ATPase.. J Biol Chem.

[27] Seeley ES, Kato M, Margolis N, Wickner W, Eitzen G (2002). Genomic analysis of homotypic vacuole fusion.. Mol Biol Cell.

[28] Obara K, Kojima R, Kihara A (2013). Effects on vesicular transport pathways at the late endosome in cells with limited very long-chain fatty acids.. J Lipid Res.

[29] Reinke A, Chen JC, Aronova S, Powers T (2006). Caffeine targets TOR complex I and provides evidence for a regulatory link between the FRB and kinase domains of Tor1p.. J Biol Chem.

[30] Dubouloz F, Deloche O, Wanke V, Cameroni E, De Virgilio C (2005). The TOR and EGO protein complexes orchestrate microautophagy in yeast.. Mol Cell.

[31] Smindak RJ, Heckle LA, Chittari SS, Hand MA, Hyatt DM, Mantus GE, Sanfelippo WA, Kozminski KG (2017). Lipid-dependent regulation of exocytosis in S. cerevisiae by OSBP homolog (Osh) 4.. J Cell Sci.

[32] Tian S, Ohta A, Horiuchi H, Fukuda R (2018). Oxysterol-binding protein homologs mediate sterol transport from the endoplasmic reticulum to mitochondria in yeast.. J Biol Chem.

[33] Faulhammer F, Kanjilal-Kolar S, Knödler A, Lo J, Lee Y, Konrad G, Mayinger P (2007). Growth control of Golgi phosphoinositides by reciprocal localization of sac1 lipid phosphatase and pik1 4-kinase.. Traffic.

[34] Foti M, Audhya A, Emr SD (2001). Sac1 lipid phosphatase and Stt4 phosphatidylinositol 4-kinase regulate a pool of phosphatidylinositol 4-phosphate that functions in the control of the actin cytoskeleton and vacuole morphology.. Mol Biol Cell.

[35] Lipp NF, Gautier R, Magdeleine M, Renard M, Albanèse V, Čopič A, Drin G (2019). An electrostatic switching mechanism to control the lipid transfer activity of Osh6p.. Nat Commun.

[36] Rossanese OW, Reinke CA, Bevis BJ, Hammond AT, Sears IB, O'Connor J, Glick BS (2001). A role for actin, Cdc1p, and Myo2p in the inheritance of late Golgi elements in Saccharomyces cerevisiae.. J Cell Biol.

[37] Arai S, Noda Y, Kainuma S, Wada I, Yoda K (2008). Ypt11 functions in bud-directed transport of the Golgi by linking Myo2 to the coatomer subunit Ret2.. Curr Biol.

[38] Costanzo M (2016). A global genetic interaction network maps a wiring diagram of cellular function.. Science.

[39] Surma MA, Klose C, Peng D, Shales M, Mrejen C, Stefanko A, Braberg H, Gordon DE, Vorkel D, Ejsing CS, Farese R, Simons K, Krogan NJ, Ernst R (2013). A lipid E-MAP identifies Ubx2 as a critical regulator of lipid saturation and lipid bilayer stress.. Mol Cell.

[40] Aronova S, Wedaman K, Anderson S, Yates J, Powers T (2007). Probing the membrane environment of the TOR kinases reveals functional interactions between TORC1, actin, and membrane trafficking in Saccharomyces cerevisiae.. Mol Biol Cell.

[41] Casler JC, Glick BS (2020). A microscopy-based kinetic analysis of yeast vacuolar protein sorting.. Elife.

[42] Ha SA, Torabinejad J, DeWald DB, Wenk MR, Lucast L, De Camilli P, Newitt RA, Aebersold R, Nothwehr SF (2003). The synaptojanin-like protein Inp53/Sjl3 functions with clathrin in a yeast TGN-to-endosome pathway distinct from the GGA protein-dependent pathway.. Mol Biol Cell.

[43] Ma M, Kumar S, Purushothaman L, Babst M, Ungermann C, Chi RJ, Burd CG (2018). Lipid trafficking by yeast Snx4 family SNX-BAR proteins promotes autophagy and vacuole membrane fusion.. Mol Biol Cell.

[44] Tani M, Kuge O (2012). Involvement of complex sphingolipids and phosphatidylserine in endosomal trafficking in yeast Saccharomyces cerevisiae.. Mol Microbiol.

[45] Wu Y, Takar M, Cuentas-Condori AA, Graham TR (2016). Neo1 and phosphatidylethanolamine contribute to vacuole membrane fusion in Saccharomyces cerevisiae.. Cell Logist.

[46] Tedrick K, Trischuk T, Lehner R, Eitzen G (2004). Enhanced membrane fusion in sterol-enriched vacuoles bypasses the Vrp1p requirement.. Mol Biol Cell.

[47] Reece-Hoyes JS, Walhout AJM (2018). High-Efficiency Yeast Transformation.. Cold Spring Harb Protoc.

[48] Voth WP, Jiang YW, Stillman DJ (2003). New 'marker swap' plasmids for converting selectable markers on budding yeast gene disruptions and plasmids.. Yeast.

[49] Tang F, Kauffman EJ, Novak JL, Nau JJ, Catlett NL, Weisman LS (2003). Regulated degradation of a class V myosin receptor directs movement of the yeast vacuole.. Nature.

