# Supplementary material for: Up-regulation of Osh6 boosts an anti-aging membrane trafficking pathway toward vacuoles
Source: Microb Cell. 2022 Jul 15;9(8):145–57. doi: 10.15698/mic2022.08.783 (PMC9344199; doi:10.15698/mic2022.08.783)
Supplement: Supplementary file 1 [file mic-09-145-s01.pdf]

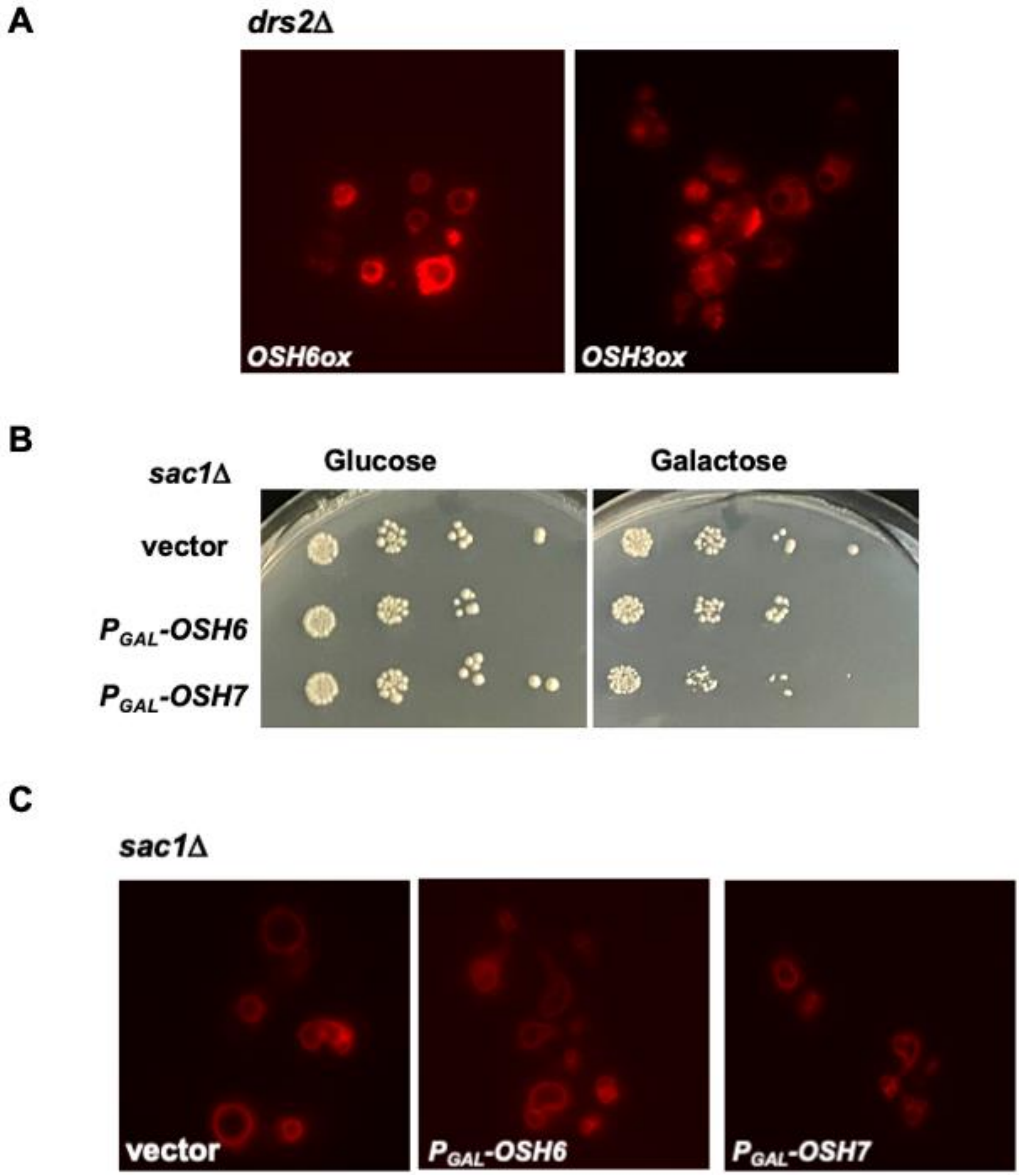

FIGURE S1: *OSH6* differentially interacts with *DRS2* and *SAC1*. (A) Vacuolar morphology of *drs2*Δ cells with a high copy *OSH6* (pCB237) or *OSH3* (pCB238) plasmid. **(B)** Growth of *sac1*Δ cells with the indicated plasmid on SC-URA with glucose or galactose media at 30°Cfor two days. **(C)** Vacuolar morphology of *sac1*Δ cells with the indicated plasmid.

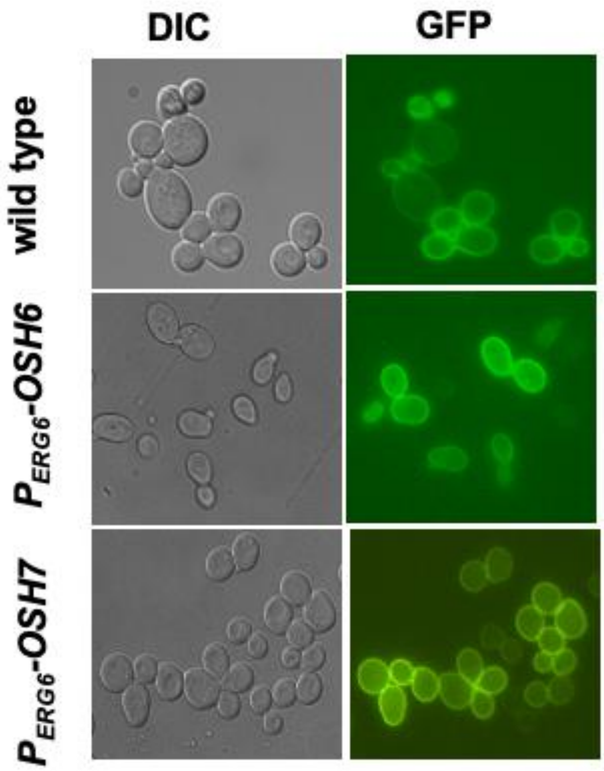

FIGURE S2: Localization of phosphatidylserine. Cells of the indicated strains were transformed with the PS-labeling LactC2-GFP plasmid. The transformants were grown to early log phase and then photographed.

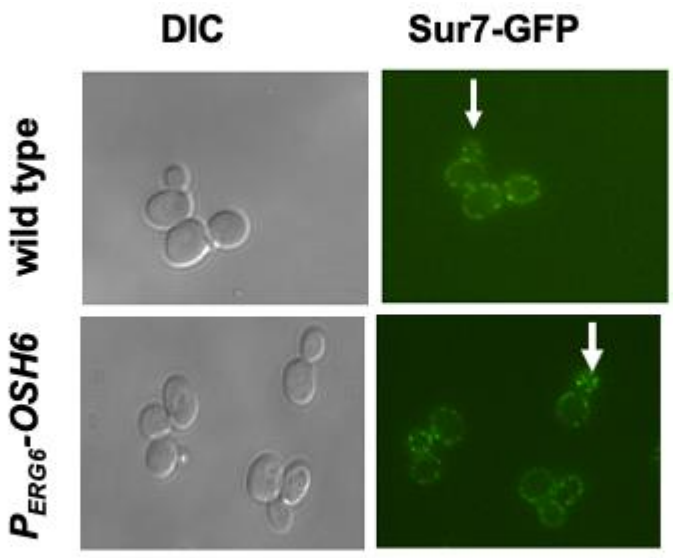

FIGURE S3: Over-expression of *OSH6* does not alter Sur7 secretion. Cells of a wild type strain carryingSur7-GFP (DLY046) and a P_{ERG6}-*OSH6* strain carrying the Sur7-GFP (FTY515) were grown in synthetic complete medium to early log phase and then photographed. The arrow points to a small bud of P_{ERG6}-*OSH6* cells with Sur7 on the bud plasma membrane.

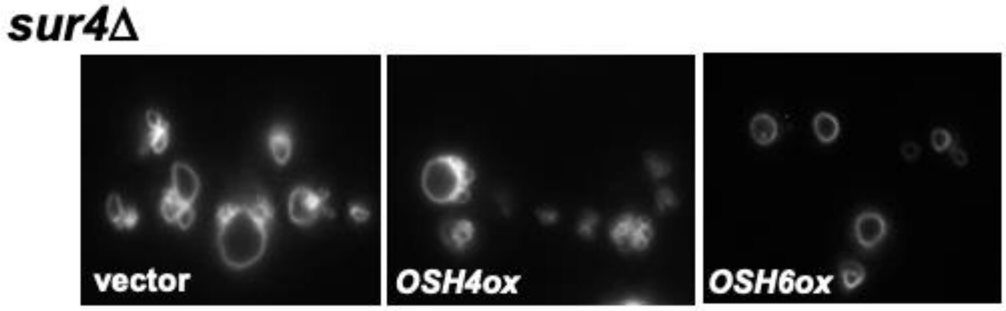

FIGURE S4: Over-expression of *OSH6* promotes vacuole fusion in *sur4*Δ. Cells of *sur4*Δ were transformed with the indicated plasmids, grown to early log phase, labeled by FM4-64 for one hour and chased for three hours at 30°C, and then photographed.

TABLE S1. Strains used in this study.

Strain	Genotype	Source	Used in
BY4742	MATα *his3Δ1 leu2Δ0 lys2Δ0 ura3Δ0*	Invitrogen	Figs. 2A, B, C, 5B, 6C, S2
FTY536	BY4742 *URA3*::*KanMx*-P$_{ERG6}$-*OSH6*	this study	Figs. 2A, B, C, S2
FTY521	BY4742 *URA3*::*KanMx*-P$_{ERG6}$-*OSH7*	this study	Figs. 2A, B, S2
FTY624	BY4742 *URA3*::*SEC7-mCerry*-P$_{ERG6}$-*OSH6*	this study	Fig. 2D
FTY625	BY4742 *URA3*::*SEC7-mCerry*-P$_{ERG6}$-*OSH7*	this study	Fig. 2D
drs2Δ	BY4742 *DRS2*::*KanMx*	Invitrogen	Figs. 1, S1A
sac1Δ	BY4742 *SAC1*::*KanMx*	Invitrogen	Fig. 2, S1B, C
DLY046	BY4742 *SUR7*-GFP::*KanMX*	[19]	Fig. S3
FTY515	BY4742 *URA3*- P$_{ERG6}$-*OSH6 SUR7-GFP-KanMx*	this study	Fig. S3
QAY559	BY4742 *PMA1-mCherry-HIS3Mx*	[19]	Figs. 3A, 4A
FTY518	BY4742 *URA3*-P$_{ERG6}$-*OSH6 PMA1-mCherry-HIS3Mx*	this study	Figs. 3A, 4B
HHY103	BY4742 *PMA1-GFP-hphNT1*	[12]	Fig. 3B
FTY517	MATa *his3Δ1 leu2Δ0 lys2Δ0 ura3Δ0 URA3*-P$_{ERG6}$-*OSH6 PMA1-mCherry-HIS3Mx*	this study	Fig. 3B
lag1Δ	BY4742 *LAG1*::*KanMx*	Invitrogen	Fig. 6
FTY373	BY4742 *URA3*-P$_{ERG6}$-*OSH6*	[5]	Fig. 6C
FTY 527	BY4742 *URA3*-P$_{ERG6}$-*OSH6 LAG1*::*KanMx*	this study	Fig. 6C
FTY 528	BY4742 *URA3*-P$_{ERG6}$-*OSH6 PMA1-mCherry-HIS3Mx LAG1*::*KanMx*	This study	Fig 5A
vps13Δ	BY4742 *VPS13*::*KanMx*	Invitrogen	Fig. 6C
FTY534	BY4742 *URA3*-P$_{ERG6}$-*OSH6 VPS13*::*KanMx*	this study	Fig. 6C
FTY532	QAY559 *VPS13*::*KanMx*	this study	Fig. 5
FTY541	BY4742 *URA3*-P$_{ERG6}$-*OSH6 VPS13*::*KanMx PMA1-mCherry-HIS3*	this study	Fig. 5
gga2Δ	BY4742 *GGA2*::*KanMx*	Invitrogen	Fig. 5B
sur4Δ	BY4742 *SUR4*::*KanMx*	Invitrogen	Fig. S4
560	BY4742 *GGA2*::*KanMx PMA1-mCherry-HIS3*	this study	Fig. 5B
562	BY4742 *URA3*-P$_{ERG6}$-*OSH6 GGA2*::*KanMx*	this study	Fig. 5B
564	BY4742 *URA3*-P$_{ERG6}$-*OSH6 GGA2*::*KanMx PMA1-mCherry-HIS3*	this study	Fig. 5B
